# The Approaching Election

**Published:** 1906-01-06

**Authors:** 


### The Hospital.

A Journal of

The Medical Sciences and Hospital Administration.

Vol. XXXIX.—No. 1,006. SATURDAY, JANUARY 6, 1906.

### THE APPROACHING ELECTION.

The first noteworthy event of the year upon which we are entering seems likely to be the election of a new House of Commons; and at such a time it is perfectly legitimate for the members of all professions or callings to bethink them of their own special needs or requirements; and, so long as these are kept in due subjection to larger national interests, to seek to bring them to the notice of the candidates who solicit their suffrages. The reservation mentioned above is necessary; because nothing can be more opposed to genuine patriotism, or to any real desire for the welfare of the community as a whole, than the endeavours, most frequently made at by-elections, to render the result dependent upon some side issue, or upon some trumpery fad which may have local supporters or opponents. On the present occasion it is not difficult to perceive that the constituencies will be appealed to mainly on the questions of Home Rule for Ireland and of such fiscal reform as may at the same time encourage home industries and draw closer the bonds of Empire; and that to these all others will be for the time subordinate. Important, but secondary, positions will be held by education, licensing reform, and the relief or prevention of destitution; and it may reasonably be feared, in view of the experience of past Parliaments, that discussions on these subjects will be so conducted as to involve the lapse of much more time than would really be needed for considering them, and to afford unlimited opportunities for obstruction and delay. Louis XVI. was accustomed to use the single word "*rien*" as a favourite entry in his diary, even at times which were deciding the fate of himself and of his dynasty, and it would frequently be sufficient to express the actual work of the House of Commons, on days when measures are being neglected which would tend to the amelioration or removal of many evils by which large classes of the nation are more or less considerably affected. Party conflicts on party questions are manifest necessities of the period in which we live, and of the system upon which we are governed; but they might be conducted in a manner sufficiently business-like to leave time for due consideration of subjects upon which men of all parties might agree, and from the neglect of which men of all parties are equally liable to suffer. We are far from supposing that such are exclusively medical in their nature, or that the members of other callings might not have important reforms to urge. Even the Law, in spite of its abundant Parliamentary representation, might no doubt be amended in the interest of practitioners as well as of clients; and it would be difficult to discover anyone who is entirely satisfied with the state of any of the public services. Notwithstanding the just demands of others, our own minds are naturally most alive to the requirements which fall most within the ken of our own experience; and we would strongly urge upon medical electors that they should lose no opportunity of seeking to impress upon the flexible minds of candidates the amount of injury to the public that is wrought or permitted by defects of legislation which it would not be a matter of great difficulty to remove.

Among questions of this kind, perhaps the first place should be given to the enormous amount of preventable sickness and mortality which is directly occasioned, in almost all parts of the kingdom, by the systematic neglect of local authorities to exercise the powers which have been entrusted to them by the Legislature for the express purpose of preventing the evils which they continue to permit. The stereotyped answer to this complaint is, of course, that the local authorities in question are elected by the communities living under their government, and that, unless these communities were satisfied, they would elect others. The answer deceives nobody, heals no sickness, and has not on any single occasion brought the dead to life. Everybody knows that the duties of local authorities are in large measure sanitary, and that the questions which determine the elections are in large measure political. When this is the case, elections can only be influenced by organisations; but the sufferers from neglect of sanitation are mainly the poor, and they cannot organise. Moreover, they are usually too ignorant to be aware of the causes of their sufferings. The local authorities knowingly permit the continuance of a polluted water supply; and, some day, a tramp comes along and adds the specific microbe of typhoid fever to the pollution which is customary. The mayor and councillors take alarm at the appearance of the first cases; and no water from the polluted source is used in their households until it has been boiled. The poor sicken by hundreds and die by scores; while the

"authorities" to whose neglect the epidemic is due sit at home and congratulate themselves upon the foresight which has preserved their own families. In view of the frequency of such occurrences, sometimes on a great scale, more often, of course, on a small one which does not attract public notice, it would surely be useful if medical electors would call the attention of candidates to the notorious facts, and would ask them to promote a measure which should give to any ratepayer an adequate power of appeal against the neglects or misdoings of the "authority" under which it may be his fate to live and suffer. In the particular case supposed, the epidemic is in time brought to a conclusion, and the amount of injury which it has wrought, either by causing death or by weakening survivors, can to some extent be estimated. But other forms of neglect, although perhaps less conspicuous at any given moment, are possibly of still greater permanent importance. The terrible state of many urban habitations is undoubtedly the chief cause of the alleged deterioration of physique among large numbers of the lower industrial classes, leading, as it does, to immorality, to dirt, to foul air, to the multiplication of vermin, to overcrowding, to defective ventilation, and to the spread of various contagious diseases. It is largely dependent upon neglect of powers which local authorities have long possessed, but which, for various reasons, they have been unwilling to exercise. England has too long been "expecting" municipalities to do their duty; and the time has surely come when expectation should be reinforced by compulsion in cases where it would otherwise be disappointed.

